# Development of a brief core set for knee dysfunction based on the International Classification of Functioning, Disability and Health: assessing construct validity and measurement potential

**DOI:** 10.1186/s12891-024-07635-3

**Published:** 2024-07-03

**Authors:** Andersom Ricardo Fréz, Geide Rosa Coelho, Bruno de Barros Pereira, Aline Cristiane Binda, Cristina Maria Nunes Cabral

**Affiliations:** 1 Physical Therapy Department, Universidade Estadual do Centro-Oeste, Alameda Élio Antonio Dalla Vecchia, 838, Guarapuava, Paraná 85040-167 Brazil; 2 Master’s and Doctoral Program in Physical Therapy, Universidade Cidade de São Paulo, São Paulo, Brazil; 3 Master’s and Doctoral Program in Physics Teaching, Universidade Federal do Espírito Santo, Vitória, Brazil

**Keywords:** ICF, Core set, Knee, Health evaluation, Disability evaluation, Construct validity

## Abstract

**Background:**

The comprehensive core set for knee dysfunction was developed to classify the functioning of people with any knee dysfunction. To be used as a clinical instrument to measure the functioning of people with knee dysfunction, the construct validity of the core set still needs to be assessed. The purpose of this study was to analyze the construct validity of the comprehensive core set for knee dysfunction as an instrument to measure functioning.

**Methods:**

A cross-sectional study with 200 participants with knee dysfunction with or without clinical diagnosis of knee pathology, with or without complaint of pain, with or without instability, and/or with or without knee movement restriction of any type. Participants were assessed using the comprehensive core set for knee dysfunction with 25 categories, the subjective form from the International Knee Documentation Committee scale, and measures of self-perceived general health and functioning. The construct validity of the core set was assessed by Rasch analysis, and the external construct validity was assessed by correlation between the score of the brief core set for knee dysfunction with the subjective form from the International Knee Documentation Committee scale, and scores of self-perception of health and functioning.

**Results:**

Twelve categories were consistent with a unidimensional construct, with no difference in the response pattern for age, sex, educational level, and time of complaint. These categories were included in the brief core set for knee dysfunction. The mean score of the brief core set was 37 ± 21 points, a value classified as moderate impairment regarding functioning. Correlations with the subjective form from the International Knee Documentation Committee scale and scores of self-perception were adequate (*p* < 0.01; *r* > 0.5).

**Conclusion:**

The brief core set for knee dysfunction, a set with 12 categories, can be used as a clinical instrument to measure and score the functioning of people with knee dysfunction, aged between 18 and 89 years, with adequate construct validity.

**Supplementary Information:**

The online version contains supplementary material available at 10.1186/s12891-024-07635-3.

## Background

Knee dysfunction is a complex interaction of multiple factors [1]. Knee dysfunction can be due to a pathological process at the knee joint [1], or changes in body functions, activities, and social participation [2]. Previous injuries or knee structure changes are not always observed [2]. Since knee dysfunction can lead to limitations and disabilities that negatively impact quality of life and functioning during activities of daily living [3], it is important to assess the functioning of patients using an instrument with a comprehensive perspective, as recommended by the International Classification of Functioning, Disability and Health (ICF).

The ICF was developed to broaden the perspective on people’s functioning, including components such as body functions and structures, activities, participation, environmental, and personal factors [4]. To increase the viability and operational use of the ICF, the core sets project was created [5, 6]. The purpose of a core set is to establish a selection of the ICF categories that describe the functioning of a person in a specific health condition or in a specific care context [6]. This selection and subsequent reduction in the number of categories in the core sets, as opposed to the use of the entire ICF for the assessment of a health condition, encourages clinical use [5, 6].

A preliminary core set for knee dysfunction with 24 categories capable of assessing the functioning and health of people with knee dysfunction was created based on the opinion of patients and then using a statistical model by regression analyses [7]. Afterwards, a panel of experts participated in the content validity assessment of this preliminary core set [8]. After a 4-round Delphi, 25 categories showed adequate content validity to compose the comprehensive core set for knee dysfunction [8]. This comprehensive core set indicates the main ICF categories that should be considered when assessing patients with knee dysfunction [8].

The ICF is based on a conceptual model in which functioning is an umbrella term for body functions and structures as well as for activities and participation [4, 9, 10]. A clinical measure of functioning based on the integration of information obtained from an ICF core set can be considered a unidimensional model [9–11]. Rasch analysis allows this unidimensional analysis by checking the data variance, which is the sum of the squares of the values predicted by the analysis around their central values [12]. Rasch analysis can also test whether the order of qualifiers in the comprehensive core set for knee dysfunction is adequate to represent the degree of knee dysfunction [13]. In addition, the consistency between factors such as age, sex, educational level, and time of complaint in the core set [14] allows comparing the functioning in different contexts and populations. Therefore, if the categories of the comprehensive core set for knee dysfunction reflect a unidimensional instrument for assessing body functions and structures, activities and participation, and environmental factors and if the categories are consistent in populations, well-directed, and not redundant, this core set can become a clinical instrument to measure the functioning of people with any knee dysfunction.

Thus, the primary objective of this study was to analyze the construct validity of the comprehensive core set for knee dysfunction as a potential instrument for measuring functioning. The secondary objective was to verify whether there is a difference in the construct validity of the comprehensive core set for knee dysfunction when applied during an interview (conducted by a person trained in the use of the ICF) or self-administered.

## Methods

### Study design

Cross-sectional study to assess construct validity. All methods were carried out in accordance with relevant guidelines and regulations.

### Setting

Face-to-face data collection with an interview was carried out at all levels of care. The interview settings were: Primary Health Care Unit of Bonsucesso (Guarapuava, Paraná), Physical Therapy School of Universidade Estadual do Centro-Oeste (Guarapuava, Paraná), Center for Excellence in Clinical Research in Physical Therapy at Universidade Cidade de São Paulo (São Paulo, São Paulo), and São Vicente de Paulo Charity Hospital (Guarapuava, Paraná). Due to the Covid-19 pandemic, which resulted in social distancing, remote assessments were carried out with a self-administered questionnaire in all levels of care and in several cities in Brazil.

### Participants

Participants were aged 18 years or older and had subjective complaints of pain, instability, and/or knee movement restriction of any type. Clinical diagnosis of knee pathology and undergoing treatment were not mandatory for inclusion, as well as having prior surgery was not considered for exclusion. Exclusion criteria were lower limb amputation and congenital and/or acquired malformation distal to the knee. Eligibility was assessed by a standardized set of questions.

Two hundred urban participants were recruited and assessed: 100 were recruited by verbal invitation at the study settings for face-to-face interview assessments, and 100 were identified in social networks of associations of physiotherapists and groups of professionals involved with knee rehabilitation, and in the general community for the remote assessments with a self-administered questionnaire. The assessments were conducted from October 2019 to June 2020.

### Variables and measurement

Sample characteristics were assessed with a questionnaire including questions on sociodemographic details and clinical conditions. After that, the comprehensive core set for knee dysfunction was applied using the description of each category as a question and the qualifiers as answer options. The comprehensive core set for knee dysfunction had 25 categories, including 11 categories from the component body functions, three representing body structures, 10 activities and participation, and one environmental factor [8]. The qualifiers for the components body functions, body structures, and activities and participation had five possible answers, ranging from 0 to 4, with 0 being no problem and 4 being a major problem. Additional qualifiers for the body structures and activities and participation components were not used. The qualifiers for the environmental factors component had nine possible answers, ranging from facilitators (+ 1 to + 4, mild facilitator to complete facilitator, respectively) to barriers (1 to 4, mild barrier to complete barrier, respectively) or no barriers/no facilitators (qualifier 0). For all components, qualifier 8 meaning “not specific” and qualifier 9 meaning “not applicable” could also be used [4].

The subjective knee assessment form of the International Knee Documentation Committee (IKDC) [15] was also applied. The IKDC had 10 questions that evaluate symptoms, sports activities, and functioning. The total score ranged from 0 to 100, in which 100 indicated that the patient had no limitations with daily living or sports activities, as well as the absence of symptoms [16].

Finally, to assess self-perception of general health and functioning, patient answered two questions: one on general health status and one on functioning, using a numerical scale from 0 to 10 as answers, in which 0 meant poor general health and/or functioning, and 10 meant excellent general health and/or functioning [17].

### Procedures

For the face-to-face interviews, patients and their caregivers who met the eligibility criteria were approached and assessed. Clinical and resident physiotherapists previously trained in the use of the ICF conducted the interview by applying the sociodemographic and clinical questionnaire and the measurement instruments (comprehensive core set for knee dysfunction [8], IKDC [16], and self-perception of general health and functioning [17]). For remote assessments, the same questionnaires and instruments were sent via an online form for the participants recruited via social networks. The online form was assessed through a link and had an initial invitation text with the eligibility criteria to participate in the study and the informed consent form. Additionally, the form was answered by the participants themselves without an interview.

### Statistical analysis

Descriptive statistics were used to characterize the sample. The sociodemographic and clinical characteristics of the participants assessed by a face-to-face interview and a self-administered questionnaire were compared. For continuous data with normal distribution, the t-test for unpaired data was applied. For data with non-normal distribution, the Mann-Whitney test was applied. For categorical data, the chi-square test was applied. For these analyses, Statistical Package for the Social Sciences version 23.0 was used, and *p* < 0.05 was considered significant.

Rasch model was used to examine whether the categories of the comprehensive core set for knee dysfunction constitute a unidimensional instrument with adequate construct validity to assess the functioning of people with knee dysfunction. For this analysis, Winsteps version 4.5.5 was used.

For all components, the qualifiers ranged from 0 to 4. To make this ordinal scale feasible for the environmental factors component, the qualifiers that indicated a facilitator were recoded as the qualifier 0 (no barriers). As qualifiers 8 and 9 were not integrated into the ordinal scale of qualifiers, they were considered as missing values, because Rasch model allowed analysis with missing values [18, 19].

The construct validity for the components body functions and structures, activities and participation, and environmental factors was assessed using Rasch model for polytomous categories. The Partial Credit Model was applied, which was valid without the assumption of equidistance between limits between the categories of the ICF [20]. For this study, the variable “person” was the study participant, while the variable “item” was the ICF category.

Initially, the separation coefficient between the participants and the categories was calculated. This coefficient is analogous to Cronbach’s alpha, a statistical calculation that determines the scale’s reliability level [21]. In addition, the separation coefficient between the participants produces a less misleading estimate, because Cronbach’s alpha always exceeds the maximum possible value for the reliability of the measures resulting from Rasch model [12].

The two primary fit statistics for Rasch model were considered: the information-weighted (or inlier-sensitive) fit statistic (infit), which was more sensitive to responses in the categories close to the participants’ functioning levels, and the outlier sensitive fit statistic (outfit) which was more influenced by responses in the categories that were lighter or more severe than the participant’s functioning [22, 23]. Thus, these statistical calculations quantified residues (MnSq) for the items in relation to the model [12, 23]. The ideal adjustment value for MnSq infit or MnSq outfit was 1, with values between 0.50 and 1.50 being considered acceptable [12]. MnSq values greater than 1.5 indicated that the qualifiers in these categories were unpredictable or erratic. In other words, unexpectedly participants with lower severity were identified with greater severity in one category or vice versa. This indicated that the category did not match the other categories to define a continuum as far as functioning is concerned or that there were problems in its definition, requiring revision. In contrast, MnSq values less than 0.5 indicated little variability of qualifiers in that category, that is, the response pattern was predictable or deterministic. The first result represented a major threat to construct validity; the second indicated that the item did not differentiate people with different functioning levels, contributing little to the definition of the construct [24].

To assess the unidimensional characteristics of the core set for knee dysfunction, we verified whether the first Rasch dimension explained most of the data variance, considering the functioning classified by the ICF. To guarantee the unidimensional characteristics of the scale, the variance explained by the measure in the first dimension should be greater than 50% (R^2^ > 0.50) [12]. Correlations between residues of items with r above 0.30 were also assessed, which indicated local dependence and possible violations of unidimensional characteristics [25].

The scale for measures of functioning and condition severity of the ICF categories was a scale of logits, which ranged from − 3.00 to + 3.00 [23]. The estimates of difficulty of each item in Rasch model had their average set at 0, with negative values indicating that the category was easier (representing a less intense level of the construct continuum) than the average of the set of categories and conversely, a category with a positive logit was more difficult than the set average [23]. Thus, the greater the participant’s functioning impairment was, the greater their likelihood of receiving high qualifiers in all categories, easy or difficult. The easier the category was, the more likely the person was to receive a low qualifier. When all items in a test met these expectations, it meant that the test fitted the model, and the likelihood was that participants with more functioning had lower scores than those with less functioning [24].

A pattern of uniform responses when considering variation in age, sex, educational level, and time of complaint was also assessed using analyses of Differential Item Functioning (DIF). The bias in the items of the instruments, known as DIF, occurred when there was a difference in the probability of response to an item for two individuals belonging to different population groups (for example, men and women), but who had the same level in the continuum of the measure [26]. Thus, the presence of DIF was indicative that the variation in the qualifiers was not only influenced by the construct investigated by the instrument, but also by a second characteristic, which interacted with the property that was the target of the evaluation [27]. DIF was an undesirable aspect because it could produce differences in meaning between the scores of different population groups, even when there were no real differences, underestimating or overestimating the assessed category [28]. DIF was assessed by analyzing the variance of each category, thus comparing the scores according to age, sex, educational level, and time of complaint [14]. For these analyses, age and time of complaint were dichotomized below and above the mean and median, respectively. The educational level was also dichotomized into below and above 11 years of study, considering high school completion as a cut-off point. We compared the differences in the distribution of responses in each category between groups (face-to-face and remote assessment). The Mantel-Haenszel chi-square was used to test the statistical significance of this difference. We opted for a conservative significance level of *p* < 0.01, due to the large number of statistical tests without previous hypotheses, which increased the chance of type I error. The categories that presented adequate results in all steps of the analysis were included in the brief core set for knee dysfunction, which could be considered as a measuring tool.

Finally, the external validity of the construct was determined by the correlation of the brief core set for knee dysfunction with the IKDC and with the measures of self-perception of general health and functioning. To that end, we created a score calculated by adding the qualifier scores and dividing it by the number of categories answered and multiplying the result by 25. Thus, the total score varied from 0 to 100, allowing the percentage of the ICF qualifiers to be used as a reference [4]. Scores ranging from 0 to 4 indicated that there was no impairment in functioning, scores from 5 to 24 indicated mild impairment of functioning, scores from 25 to 49 indicated moderate impairment in functioning, scores from 50 to 95 indicated severe impairment in functioning, and scores above 96 indicated total impairment in functioning. Data with normal distribution were analyzed using Pearson’s correlation coefficient and data with non-normal distribution were analyzed using Spearman’s correlation coefficient. Given that the IKDC and the self-perception of functioning measured a construct similar to the core set, a negative correlation with IKDC ≥ 0.50 was considered adequate, and a positive correlation with the measure of self-perception of functioning ≥ 0.50 was considered adequate. For the measure of self-perception of general health, a negative correlation with the brief core set for knee dysfunction between 0.30 and 0.50 was considered appropriate, as they measure different, but related constructs [29].

## Results

Table 1 presents the clinical and sociodemographic characteristics of the patients. As the groups showed significant differences in most variables (except sex, knee as the most affected condition, knee surgery, and time of surgery), we decided to analyze the groups separately.

**Table 1:** Sample characteristics

Characteristics	Face-to-face sample (*n* = 100)	Remote sample (*n* = 100)	Total (*n* = 200)
Sex			
Male	36 (36.0)	29 (29.0)	65 (32.5)
Female	64 (64.0)	71 (71.0)	135 (67.5)
Age (years)	51.3 (18.4)	35.7 (14.5)^δ^	43.5 (18.3)
Educational level			
< 1 year	1 (1.0)	0 (0.0)^δ^	1 (0.5)
1–3 years	13 (13.0)	1 (1.0)	14 (7.0)
4–11 years	24 (24.0)	3 (3.0)	27 (13.5)
12–15 years	43 (43.0)	34 (34.0)	77 (38.5)
> 16 years	19 (19.0)	62 (62.0)	81 (40.5)
Current marital status			
Single	24 (24.0)	49 (49.0)^δ^	73 (36.5)
Married	53 (53.0)	45 (45.0)	98 (49.0)
Divorced	8 (8.0)	4 (4.0)	12 (6.0)
Widowed	15 (15.0)	2 (2.0)	17 (8.5)
Main work status			
Paid word	27 (27.0)	41 (41.0)^δ^	68 (34.0)
Retired	35 (35.0)	4 (4.0)	39 (19.5)
Self employed	4 (4.0)	26 (26.0)	30 (15.0)
Student	7 (7.0)	23 (23.0)	30 (15.0)
Keeping house/homemaker	15 (15.0)	2 (2.0)	17 (8.5)
Pension and disability benefits	8 (8.0)	0 (0.0)	8 (4.0)
Unemployed	4 (4.0)	3 (3.0)	7 (3.5)
Voluntary	0 (0.0)	1 (1.0)	1 (0.5)
Recruitment			
Unattended community participant	17 (17.0)	79 (79.0)^δ^	96 (48.0)
Physiotherapy clinic	52 (52.0)	10 (10.0)	62 (31.0)
Home care	14 (14.0)	3 (3.0)	17 (8.5)
Primary health care units	16 (16.0)	1 (1.0)	17 (8.5)
Telerehabilitation	0 (0.0)	6 (6.0)	6 (3.0)
Medical clinic	1 (1.0)	0 (0.0)	1 (0.5)
Hospital	0 (0.0)	1 (1.0)	1 (0.5)
Complaint			
Unilateral	58 (58.0)	60 (60.0)	118 (59.0)
Bilateral	42 (42.0)	40 (40.0)	82 (41.0)
Main knee complaint			
Pain	78 (78.0)	70 (70.0)^δ^	148 (74.0)
Instability	6 (6.0)	11 (11.0)	17 (8.5)
Crepitation	3 (3.0)	14 (14.0)	17 (8.5)
Stiffness	11 (11.0)	4 (4.0)	15 (7.5)
Muscle strength	2 (2.0)	0 (0.0)	2 (1.0)
Sensory alteration	0 (0.0)	1 (1.0)	1 (0.5)
Time of injury/complaint (months)^*^	25 (12–60)	12 (2-31.5)^δ^	24 (6–48)
IKDC (0 to 100)	62.2 (10.5)	57.0 (7.9)^δ^	59.6 (9.6)
Self-perception of general health (0 to 10)	7.3 (2.0)	8.0 (1.3)^δ^	7.7 (1.7)
Self-perception of functioning (0 to 10)	7.1 (2.2)	8.0 (1.4)^δ^	7.5 (1.9)
Diagnosis			
Osteoarthritis	35 (35.0)	7 (7.0)^δ^	42 (21.0)
Patellofemoral pain syndrome	12 (12.0)	12 (12.0)	24 (12.0)
Ligament injuries	7 (7.0)	6 (6.0)	13 (6.5)
Meniscal lesions	6 (6.0)	7 (7.0)	13 (6.5)
Rheumatoid arthritis	4 (4.0)	5 (5.0)	9 (4.5)
Patellar tendinopathy	3 (3.0)	4 (4.0)	7 (3.5)
Meniscal and ligament injuries	3 (3.0)	2 (2.0)	5 (2.5)
Osteoarthritis and ligament injuries	2 (32.0)	2 (2.0)	4 (2.0)
Femoral fractures (proximal)	3 (3.0)	0 (0.0)	3 (1.5)
Tibial fractures (diaphysis)	3 (3.0)	0 (0.0)	3 (1.5)
Patellofemoral instability	1 (1.0)	1 (1.0)	2 (1.0)
Patellofemoral instability and ligament injuries	0 (0.0)	1 (1.0)	1 (0.5)
Patellar tendinopathy and ligament injuries	0 (0.0)	1 (1.0)	1 (0.5)
Patellar ligament rupture	0 (0.0)	1 (1.0)	1 (0.5)
Femoral and patellar fractures	1 (1.0)	0 (0.0)	1 (0.5)
Femoral fracture and ligament injuries	0 (0.0)	1 (1.0)	1 (0.5)
Femoral and tibial fractures	1 (1.0)	0 (0.0)	1 (0.5)
Tibial fractures (proximal)	1 (1.0)	0 (0.0)	1 (0.5)
Tibial and patellar fractures	0 (0.0)	1 (1.0)	1 (0.5)
Patellar fractures	0 (0.0)	1 (1.0)	1 (0.5)
Genu valgum	1 (1.0)	0 (0.0)	1 (0.5)
Iliotibial band syndrome	0 (0.0)	1 (1.0)	1 (0.5)
No diagnosis/unknown	17 (17.0)	47 (47.0)	64 (32.0)
Knee surgery			
Ligamentoplasty and meniscectomy	5 (5.0)	6 (6.0)	11 (5.5)
Ligamentoplasty	5 (5.0)	4 (4.0)	9 (4.5)
Meniscectomy	3 (3.0)	2 (2.0)	5 (2.5)
Fracture fixation	5 (5.0)	0 (0.0)	5 (2.5)
Total knee arthroplasty	2 (2.0)	0 (0.0)	2 (1.0)
Arthroscopy	1 (1.0)	0 (0.0)	1 (0.5)
Patellar tenorrhaphy	1 (1.0)	0 (0.0)	1 (0.5)
No surgery	78 (78.0)	88 (88.0)	166 (83.0)
Time of surgery (months)^*a^	15 (9–60)	24 (11–105)	15 (9-67.5)
Other health problems			
1 problem	18 (18.0)	7 (7.0)^δ^	25 (12.5)
2 problems	15 (15.0)	6 (6.0)	21 (10.5)
3 problems	9 (9.0)	5 (5.0)	14 (7.0)
4 problems	7 (7.0)	4 (4.0)	11 (5.5)
≥ 5 problems	8 (8.0)	5 (5.0)	13 (6.5)
No problem	43 (43.0)	73 (73.0)	116 (58.0)

Continuous variables are expressed as mean (standard deviation) and categorical variables as absolute number (relative number). IKDC: Subjective knee assessment form of the International Knee Documentation Committee. ^δ^Significant difference between groups (*p* < 0.05), ^*^Values expressed as median and interquartile range, ^a^Variable analyzed in 34 participants.

Initially, we analyzed the construct validity of the comprehensive core set for knee dysfunction applied face-to-face via interview. After analysis of the MnSq infit and MnSq outfit, 11 categories were excluded (Appendix A). The categories b134 “sleep functions”, b260 “proprioceptive function”, b710 “mobility of joint functions”, b715 “stability of joint functions”, b760 “control of voluntary movement functions”, s7500 “structure of thigh”, s7502 “structure of ankle and foot”, d240 “handling stress and other psychological demands”, d540 “dressing”, and e150 “design, construction and building products and technology of buildings for public use” presented an unpredictability problem (MsSq infit and outfit MnSq > 1.5). Regarding the predictability of the categories (infit and outfit MnSq < 0.5), only the category d455 “moving around” was excluded. No residual correlations were observed. The categories that remained in the core set presented an adjustment of the separation reliability coefficient between participants of 0.88, which indicated high reliability. In addition, data variance explained by the measure greater than 50% was found in the first Rasch dimension (55.5%), a criterion that satisfied the unidimensional characteristic of the scale. The categories that remained in the core set and were submitted to DIF analysis are shown in Table 2.

**Table 2:** Estimates of difficulty and adjustment to Rasch model of the comprehensive core set for knee dysfunction applied face-to-face via interview conducted by a clinical or resident physiotherapist trained in the use of ICF

Person separation index	2.68			
Real RMSE (True SD)	0.42 (1.12)			
Person reliability index	0.88			
Item (category) separation index	3.74			
Real RMSE (True SD)	0.13 (0.48)			
Item (category) reliability index	0.93			
Raw variance explained by measures	55.5%			
ICF category	Measure	SE	Infit	Outfit
b235 “Vestibular functions” (balance) (*n* = 100)	0.70	0.13	0.84	0.98
b280 “Sensation of pain” (*n* = 100)	-0.88	0.11	1.33	1.39
b530 “Weight maintenance functions” (*n* = 100)	0.20	0.12	1.19	1.39
b730 “Muscle power functions” (*n* = 99)	-0.06	0.11	1.08	1.13
b770 “Gait pattern functions” (*n* = 98)	0.32	0.12	0.51	0.51
b780 “Sensations related to muscles and movement functions” (*n* = 97)	0.63	0.13	1.34	1.22
s7501 “Structure of lower leg” (*n* = 100)	-0.63	0.11	0.74	0.78
d410 “Changing basic body position” (*n* = 100)	0.20	0.12	1.16	1.01
d415 “Maintaining a body position” (*n* = 100)	-0.53	0.11	0.88	0.81
d430 “Lifting and carrying objects” (*n* = 97)	0.46	0.12	1.42	1.23
d450 “Walking” (*n* = 100)	-0.38	0.11	1.00	0.98
d470 “Using transportation” (*n* = 97)	0.63	0.13	0.62	0.59
d850 “Remunerative employment” (*n* = 49)	-0.21	0.16	1.20	1.11
d920 “Recreation and leisure” (*n* = 85)	-0.44	0.12	0.87	0.84
Mean	0.00	0.12	1.01	1.00
SD	0.50	0.01	0.27	0.26

RMSE: root mean squared error, ICF: International Classification of Functioning, Disability and Health, n: answers without the qualifiers 8 or 9, SE: standard error, SD: standard deviation.

The analysis of the response pattern of the face-to-face via interview assessments, considering the variation of age, sex, educational level, and time of complaint through DIF analyses, is shown in Table 3. Two categories showed a difference in the probability of response and were excluded from the comprehensive core set for knee dysfunction: b770 “gait pattern functions” and d450 “walking”. Thus, the brief core set for knee dysfunction with 12 categories to be applied face-to-face via interview has adequate construct validity.

**Table 3:** Differential Item Functioning of the categories of the comprehensive core set for knee dysfunction applied face-to-face via interview conducted by a clinical or resident physiotherapist trained in the use of ICF that did not present adjustment problem, Mantel-Haenszel Chi-square statistics (p value)

ICF category	Sex	Age	Educational level	Time of complaint
b235 “Vestibular functions” (balance)^a^ (*n* = 100)	0.3396	0.5238	0.9474	0.3746
b280 “Sensation of pain”^a^ (n = 100)	0.1522	0.3743	0.2874	0.2121
b530 “Weight maintenance functions”^a^ (n = 100)	0.2190	0.0802	0.2794	0.2885
b730 “Muscle power functions”^a^ (n = 99)	0.3764	0.1405	0.2392	0.3035
b770 “Gait pattern functions” (*n* = 98)	0.0070^*^	0.0927	0.8551	0.6949
b780 “Sensations related to muscles and movement functions”^a^ (n = 97)	0.4123	0.7052	0.4241	0.0592
s7501 “Structure of lower leg”^a^ (n = 100)	0.7655	0.3419	0.1364	0.4087
d410 “Changing basic body position”^a^ (n = 100)	0.4575	0.2781	0.3419	0.6347
d415 “Maintaining a body position”^a^ (n = 100)	0.2236	0.0231	0.0158	0.1848
d430 “Lifting and carrying objects”^a^ (n = 97)	0.6027	0.1088	0.0484	0.2301
d450 “Walking” (*n* = 100)	0.2873	0.3944	0.0088^*^	0.1154
d470 “Using transportation”^a^ (n = 97)	0.4702	0.9435	0.3573	0.0126
d850 “Remunerative employment”^a^ (n = 49)	0.7518	0.0952	0.4142	-
d920 “Recreation and leisure”^a^ (n = 85)	0.6251	0.2144	0.1394	0.0414

ICF: International Classification of Functioning, Disability and Health, n: answers without the qualifiers 8 or 9. ^*^Significant difference; ^a^Categories selected to compose the brief core set for knee dysfunction; - Insufficient data for analysis.

The construct validity of the comprehensive version of the remote self-administered core set for knee dysfunction was also analyzed. After the MnSq infit and MnSq outfit analysis, only the category s7502 “structure of ankle and foot” was excluded from the comprehensive core set for knee dysfunction due to an unpredictability problem (MnSq outfit > 1.5). The e150 “design, construction and building products and technology of buildings for public use” category was also excluded for showing a measure of the participants’ functioning and difficulty of the categories (logits) above 3 (Appendix B).

Despite the removal of these two categories, the core set presented a unidimensional characteristic problem, as only 42.9% of the data variance was explained by the measure. Thus, possible residual correlations (*r* > 0.30) were assessed. Two correlations were identified: between categories b235 “vestibular functions” (balance) and b760 “control of voluntary movement functions” (*r* = 0.51) and between categories b260 “proprioceptive function” and b760 “control of voluntary movement functions” (*r* = 0.46). Even after removing these categories, either alone or in combination, the comprehensive core set for knee dysfunction with a remote self-administered questionnaire still presented a unidimensional characteristic problem, because in none of the analyses the variance explained by the measure was above 43.7%, hindering the subsequent analyses.

Considering the results of the analyses separated by group, we decided not to carry out the analysis with the entire sample (*n* = 200), because the results of the analyses for the remote self-administered questionnaire were not adequate to build a measurement tool. Thus, 12 categories of the core set for knee dysfunction applied face-to-face via interview showed adequate results to compose a measurement tool. These 12 categories were considered the brief core set for knee dysfunction. Five categories belong to the body functions component, one category to the body structures component, and six categories to the activities and participation component (Table 3). This version was subjected to the external construct validity test. The qualifier scores for each participant were added up, and the total was divided by the number of categories answered and multiplied by 25. The mean score was 37 points and the standard deviation was 21 points, classified as moderate impairment in functioning, and the minimum and maximum values were 0 and 85, respectively. Adequate and significant correlations (*p* < 0.01) were observed in all analyses. The correlation with the IKDC was − 0.75 (95% confidence interval [95% CI]: -0.83 to -0.65), with the self-perception of functioning was − 0.66 (95% CI: -0.76 to -0.52), and with the self-perception of general health was − 0.60 (95% CI: -0.71 to -0.45). The brief core set for knee dysfunction with instructions for application and scoring is presented in the Appendix C.

## Discussion

This study assessed the construct validity of the comprehensive core set for knee dysfunction. The core set with a remote self-administered questionnaire did not show adequate results and should not be used. When the comprehensive core set for knee dysfunction was applied face-to-face via interview by a researcher, 12 of the 25 categories showed adequate consistency with a unidimensional construct, free of DIF for age, sex, educational level, and time of complaint, and can be used to assess functioning. Furthermore, we built an accurate and useful measurement tool based on the comprehensive core set for knee dysfunction, which included not only the symptoms and affected structure, but the body functions and activities and social participation.

The proposal for a new core set is a process with many steps that involves the participation of patients and health professionals [6]. For this reason, we decided to validate a general core set that can be used in various clinical conditions of the knee and not a core set based on a specific condition. Therefore, functioning and disability are the focus of assessment and not a particular condition. In addition, the clinical implementation of the ICF depends on the development of practical tools that allow not only classification but measurement of functioning [30].

This study used psychometric and statistical analysis to develop a stable and generalizable tool. The comprehensive core set for knee dysfunction, with 25 categories, remains an applicable instrument for clinical practice and research, as it indicates the main categories that should be considered in an assessment. The current findings suggest that the brief core set for knee dysfunction, with 12 categories, indicates which categories should be included in the calculation of a score. The other 13 categories provide extra information about the patient’s functioning, but do not add measurement accuracy. This variation in the number of categories that should be used to calculate a score was also observed in the core set for breast cancer, which has 43 categories for classification and, according to Rasch analysis, only 30 can be used as a measurement tool [31] to calculate a score.

The brief core set was based on a Rasch model that provides an interval scale and calibrates the functioning of a participant and the limitation in a category in the same analysis. Thus, through Rasch analysis, the brief core set was developed from the categories of the comprehensive core set for knee dysfunction that met all the premises of the analysis [14], based exclusively on the criteria defined for Rasch model. Although it is possible to maintain a marginally adequate or even slightly inadequate category in the analysis of construct validity for a tool, even if it affects statistical properties such as item adjustment or limit order [19], in our study we did not extrapolate the values defined for Rasch analysis. Other studies have included categories with adjustment problems during the assessment of construct validity, but authors did not justify the reason for this decision [25, 31].

The brief core set for knee dysfunction is unidimensional, indicating that a participant’s response to a category was explained by their own functional level in that category and not by other factors [32]. All categories from the brief core set for knee dysfunction were included adhering to a single construct: functioning. The brief core set also showed high scores for the separation reliability coefficient between participants and categories, which point to a tool with high reliability.

Although the ICF recognizes environmental factors as a component of the multidimensional assessment of people with some dysfunction [33], the only category in the comprehensive core set for knee dysfunction (e150 “design, construction and building products and technology of buildings for public use”) was removed from the brief version as it had an adjustment problem. For this component, an adjustment of the qualifiers to maintain the ordinal scale of the qualifiers from the other components was needed. This fact may have influenced the results because all qualifiers that indicated a facilitator were recoded as no barrier (qualifier 0). The difficulty in assessing the construct of environmental factors has already been reported in another study [14], in which authors reported that the nine levels of response do not represent a cumulative measure of the impact of these environmental factors, and it is necessary to re-code the qualifier of this component. Furthermore, there is still no consensus on the best strategy for this process of re-coding environmental factors [14, 25, 31].

Other core sets have also had construct validity assessed using Rasch analysis, such as the core sets for osteoarthritis [14], low back pain [25], breast cancer [31], and stroke [10]. The core set for generalized chronic pain had its construct validity assessed for patients with fibromyalgia [19]. One study on chronic obstructive pulmonary disease reported the construct validity of only one component of the core set [34], which had adequate construct validity for the activities and participation component. In the core set for low back pain, a self-applicable version of the activities and participation component was proposed [35], which presented adequate construct validity through Rasch analysis. Although Rasch analysis is used on a recurring basis to assess the construct validity of the ICF, there is a divergence between studies regarding the parameters adopted for the analysis, as well as for the results of the analysis that must be presented. In this study, we chose to present the measurement values (logit), infit MnSq and outfit MnSq of all categories and not just the means of the results of these analyses.

The Covid-19 pandemic brought about social isolation measures, thus limiting and subsequently preventing face-to-face evaluations via interviews. As a result, the evaluations with a remote self-administered questionnaire were carried out, and the participants rated their own functioning. It was expected that the core set could be used in this context of social restriction, however, the results were unfavorable. In the analysis of the data with a remote self-administered questionnaire, a unidimensional characteristic problem was observed, which compromised the results of the total sample. A possible explanation for this result may be how categories were presented to participants, because the online forms in which the core set categories were sent included the description of categories and what they included, without any additional information. As the ICF is written in scientific and medical terms, it may be necessary to adjust some domains to make the language easier to understand [31]. One study has already suggested developing self-administered questionnaires from a core set [36]. However, the difficulty in converting the ICF categories into a questionnaire has already been reported in the study investigating the construct validity of the core set for breast cancer [31]. In addition, a cross-cultural adaptation may be necessary to increase the understanding of categories [37]. The results of this study also highlight the importance of a trained professional in using the ICF to conduct the assessment.

As a strength of the present study, a clinical instrument to measure the functioning of people with any knee dysfunction was developed, considering the multiple aspects of functioning recommended by the ICF. As we proposed a measurement tool, a weakness of the study was the failure to evaluate other measurement properties for the instrument to have all the scientifically required parameters. The results of this study demonstrated the potential of the brief core set for knee dysfunction as a scale. Future studies can evaluate other measurement properties of the brief core set for knee dysfunction, such as inter- and intra-rater reliability and responsiveness, in addition to checking for possible ceiling and floor effects.

Regarding the limitations of this study, first, the sample was limited to only two cities in Brazil. Therefore, different results could be found in other countries or regions. Although patients were asked about other comorbidities, we did not assess whether these comorbidities impacted functioning. Furthermore, lifestyle habits and the use of orthoses were not recorded. Regarding educational level, other studies [14, 25] conducted the analysis considering the average schooling time in the country. However, in our study, educational level was assessed as a categorical variable, not allowing analysis by the average schooling time of the Brazilian population, which is 9.4 years [38]. Finally, the face-to-face sample may not represent all people with knee dysfunction, because they are relatively less educated, older, and retired. Although the study identifies which categories can be used as a measurement tool, scores from qualifiers can be considered provisional until repeated evidence in larger samples supports the current interpretation.

## Conclusion

From the comprehensive core set for knee dysfunction, 12 categories showed adequate construct validity to compose the brief core set for knee dysfunction to be applied face-to-face via interview by a professional. These 12 categories cover body functions and structures and activities and participation and can be used to measure the functioning of people with any knee dysfunction, aged between 18 and 89 years.

### Electronic supplementary material

Below is the link to the electronic supplementary material.

Appendix A

Appendix B

Appendix C

## Data Availability

The datasets used and/or analysed during the current study are available from the corresponding author on reasonable request.

## Abbreviations

ICF: International Classification of Functioning, Disability and Health
IKDC: International Knee Documentation Committee
Infit: Information-weighted (or inlier-sensitive) fit statistic
Outfit: Outlier sensitive fit statistic
MnSq: Mean-square
DIF: Differential item functioning
