# Supplementary material for: Development of a brief core set for knee dysfunction based on the International Classification of Functioning, Disability and Health: assessing construct validity and measurement potential
Source: BMC Musculoskelet Disord. 2024 Jul 3;25:512. doi: 10.1186/s12891-024-07635-3 (PMC11221104; doi:10.1186/s12891-024-07635-3)
Supplement: Supplementary file 1 — Appendix A [file 12891_2024_7635_MOESM1_ESM.pdf]

Appendix A. Estimates of difficulty and adjustment to Rasch model of the comprehensive core set for knee dysfunction applied face-to-face via interview conducted by a clinical or resident physiotherapist trained in the use of ICF

Table 1. Estimates of difficulty and adjustment to Rasch model of the comprehensive core set for knee dysfunction applied face-to-face via interview conducted by a researcher trained in the use of ICF

	**MODEL 1**				**MODEL 2**				**MODEL 3**			
Person Separation Reliability Index	2.62 (0.87)				2.73 (0.88)				2.76 (0.88)			
Item Separation Reliability Index	4.49 (0.95)				4.64 (0.96)				4.69 (0.96)			
Raw variance explained by measures	47.5%				49.3%				50.3%			
**ICF Category**	**Measure**	**SE**	**Infit**	**Outfit**	**Measure**	**SE**	**Infit**	**Outfit**	**Measure**	**SE**	**Infit**	**Outfit**
b134 (n=100)	0.12	.11	1.65	1.68	0.15	.11	1.73	1.76	0.19	.11	1.79	1.81*
b235 (n=100)	0.18	.11	0.56	0.64	0.21	.11	0.59	0.68	0.25	.11	0.61	0.70
b260 (n=100)	1.06	.15	1.28	1.18	1.12	.15	1.30	1.25	1.20	.16	1.32	1.32
b280 (n=100)	-1.00	.10	1.10	1.19	-1.03	.10	1.18	1.28	-1.02	.10	1.19	1.29
b530 (n=100)	-0.19	.10	0.81	0.93	-0.18	.10	0.86	1.03	-0.14	.10	0.89	1.05
b710 (n=97)	-0.35	.10	1.36	1.18	-0.34	.10	1.41	1.21	-0.31	.10	1.51	1.29
b715 (n=100)	0.38	.11	1.75	2.95*	-	-	-	-	-	-	-	-
b730 (n=99)	-0.39	.10	0.70	0.72	-0.38	.10	0.76	0.79	-0.36	.10	0.78	0.83
b760 (n=100)	0.80	.13	0.92	1.18	0.85	.14	0.97	1.37	0.92	.14	1.03	1.44
b770 (n=98)	-0.10	.10	0.39	0.41	-0.08	.10	0.39	0.41	-0.05	.11	0.40	0.42
b780 (n=97)	0.12	.11	0.98	0.90	0.15	.11	1.04	0.93	0.19	.11	1.07	0.96
s7500 (n=100)	0.88	.14	2.08	1.69	0.93	.14	2.13*	1.71	-	-	-	-
s7501 (n=100)	-0.81	.10	0.52	0.53	-0.83	.10	0.55	0.58	-0.81	.10	0.56	0.59
s7502 (n=96)	0.69	.13	1.58	1.69	0.74	.14	1.66	1.77	0.80	.14	1.71	1.79
d240 (n=98)	0.30	.11	1.21	1.11	0.33	.12	1.27	1.18	0.38	.12	1.32	1.21
d410 (n=100)	-0.19	.10	0.94	0.83	-0.18	.10	0.93	0.82	-0.14	.10	0.97	0.85
d415 (n=100)	-0.73	.10	0.84	0.78	-0.75	.10	0.85	0.79	-0.73	.10	0.86	0.81
d430 (n=97)	0.01	.11	1.40	1.19	0.03	.11	1.42	1.19	0.06	.11	1.44	1.20
d450 (n=100)	-0.62	.10	0.82	0.79	-0.63	.10	0.84	0.82	-0.61	.10	0.86	0.83
d455 (n=100)	-0.66	.10	0.37	0.40	-0.67	.10	0.37	0.40	-0.65	.10	0.38	0.42
d470 (n=97)	0.14	.11	0.52	0.50	0.16	.11	0.52	0.51	0.21	.11	0.53	0.51
d540 (n=98)	0.45	.12	1.54	1.22	0.48	.12	1.54	1.21	0.54	.12	1.61	1.27
d850 (n=49)	-0.47	.14	0.97	0.90	-0.49	.14	1.01	0.96	-0.46	.15	1.03	0.99
d920 (n=85)	-0.69	.11	0.77	0.74	-0.70	.11	0.80	0.76	-0.67	.11	0.80	0.76
e150 (n=100)	1.06	.15	1.46	0.87	1.12	.15	1.48	0.92	1.20	.16	1.50	0.96
Mean	0.00	.11	1.06	1.05	0.00	.12	1.07	1.01	0.00	.12	1.05	1.01
SD	0.59	.02	0.45	0.53	0.62	.02	0.44	0.38	0.62	.02	0.41	0.38

Table 1. Estimates of difficulty and adjustment to Rasch model of the comprehensive core set for knee dysfunction applied face-to-face via interview conducted by a researcher trained in the use of ICF (continued)

	MODEL 4				MODEL 5				MODEL 6			
Person Separation Reliability Index	2.81 (0.89)				2.81 (0.89)				2.84 (0.89)			
Item Separation Reliability Index	4.91 (0.96)				4.98 (0.96)				5.09 (0.96)			
Raw variance explained by measures	52.6%				53.5%				54.3%			
**ICF Category**	**Measure**	**SE**	**Infit**	**Outfit**	**Measure**	**SE**	**Infit**	**Outfit**	**Measure**	**SE**	**Infit**	**Outfit**
b134 (n=100)	-	-	-	-	-	-	-	-	-	-	-	-
b235 (n=100)	0.28	.11	0.63	0.74	0.33	.12	0.67	0.80	0.37	.12	0.68	0.83
b260 (n=100)	1.27	.16	1.31	1.43	1.35	.16	1.33	1.46	1.42	.16	1.38	1.47
b280 (n=100)	-1.05	.10	1.24	1.35	-1.04	.11	1.29	1.42	-1.04	.11	1.28	1.39
b530 (n=100)	-0.14	.11	0.96	1.23	-0.10	.11	1.01	1.26	-0.07	.11	1.03	1.27
b710 (n=97)	-0.32	.11	1.53	1.31	-0.28	.11	1.56	1.31	-0.26	.11	1.67*	1.41
b715 (n=100)	-	-	-	-	-	-	-	-	-	-	-	-
b730 (n=99)	-0.36	.10	0.82	0.88	-0.33	.11	0.85	0.91	-0.31	.11	0.84	0.91
b760 (n=100)	0.98	.14	1.10	1.53	1.05	.14	1.14	1.59	1.11	.15	1.16	1.60
b770 (n=98)	-0.04	.11	0.40	0.45	0.00	.11	0.42	0.46	0.03	.11	0.43	0.47
b780 (n=97)	0.22	.12	1.15	1.04	0.26	.12	1.20	1.08	0.31	.12	1.23	1.11
s7500 (n=100)	-	-	-	-	-	-	-	-	-	-	-	-
s7501 (n=100)	-0.84	.10	0.60	0.63	-0.82	.10	0.60	0.64	-0.82	.11	0.63	0.66
s7502 (n=96)	0.84	.14	1.73	1.82*	-	-	-	-	-	-	-	-
d240 (n=98)	0.41	.12	1.40	1.29	0.46	.12	1.46	1.34	0.51	.12	1.47	1.35
d410 (n=100)	-0.14	.11	1.04	0.90	-0.10	.11	1.02	0.89	-0.07	.11	1.10	0.95
d415 (n=100)	-0.75	.10	0.89	0.83	-0.74	.10	0.89	0.83	-0.73	.11	0.90	0.84
d430 (n=97)	0.07	.11	1.46	1.22	0.12	.11	1.50	1.26	0.15	.12	1.52	1.28
d450 (n=100)	-0.63	.10	0.89	0.87	-0.61	.10	0.91	0.89	-0.59	.11	0.94	0.92
d455 (n=100)	-0.67	.10	0.41	0.45	-0.65	.10	0.41	0.46	-0.64	.11	0.41	0.46
d470 (n=97)	0.23	.12	0.54	0.52	0.28	.12	0.55	0.53	0.32	.12	0.59	0.57
d540 (n=98)	0.56	.13	1.66	1.29	0.62	.13	1.68*	1.31	-	-	-	-
d850 (n=49)	-0.48	.15	1.03	0.98	-0.48	.15	1.12	1.05	-0.45	.15	1.13	1.06
d920 (n=85)	-0.69	.11	0.82	0.79	-0.68	.11	0.86	0.82	-0.66	.12	0.85	0.81
e150 (n=100)	1.27	.16	1.47	0.93	1.35	.16	1.51	0.95	1.42	.16	1.55	0.98
Mean	0.00	.12	1.05	1.02	0.00	.12	1.05	1.01	0.00	.12	1.04	1.02
SD	0.66	.02	0.39	0.36	0.68	.02	0.37	0.33	0.70	.02	0.37	0.33

Table 1. Estimates of difficulty and adjustment to Rasch model of the comprehensive core set for knee dysfunction applied face-to-face via interview conducted by a researcher trained in the use of ICF (continued)

	**MODEL 7**				**MODEL 8**				**MODEL 9**			
Person Separation Reliability Index	2.82 (0.89)				2.85 (0.89)				2.85 (0.89)			
Item Separation Reliability Index	5.29 (0.97)				5.14 (0.96)				4.66 (0.96)			
Raw variance explained by measures	55.8%				56.1%				55.4%			
**ICF Category**	**Measure**	**SE**	**Infit**	**Outfit**	**Measure**	**SE**	**Infit**	**Outfit**	**Measure**	**SE**	**Infit**	**Outfit**
b134 (n=100)	-	-	-	-	-	-	-	-	-	-	-	-
b235 (n=100)	0.37	.12	0.70	0.83	0.44	.12	0.73	0.88	0.55	.12	0.76	0.92
b260 (n=100)	1.46	.16	1.47	1.52	1.55	.17	1.46	1.61*	-	-	-	-
b280 (n=100)	-1.10	.11	1.33	1.44	-1.06	.11	1.32	1.42	-0.99	.11	1.31	1.39
b530 (n=100)	-0.09	.11	1.09	1.32	-0.02	.11	1.12	1.38	0.07	.11	1.15	1.40
b710 (n=97)	-	-	-	-	-	-	-	-	-	-	-	-
b715 (n=100)	-	-	-	-	-	-	-	-	-	-	-	-
b730 (n=99)	-0.34	.11	0.88	0.96	-0.28	.11	0.94	1.07	-0.19	.11	0.99	1.09
b760 (n=100)	1.14	.15	1.20	1.63*	-	-	-	-	-	-	-	-
b770 (n=98)	0.02	.11	0.45	0.48	0.08	.12	0.46	0.48	0.18	.12	0.49	0.51
b780 (n=97)	0.31	.12	1.25	1.12	0.38	.12	1.25	1.12	0.48	.12	1.29	1.16
s7500 (n=100)	-	-	-	-	-	-	-	-	-	-	-	-
s7501 (n=100)	-0.87	.11	0.66	0.68	-0.82	.11	0.68	0.71	-0.74	.11	0.70	0.72
s7502 (n=96)	-	-	-	-	-	-	-	-	-	-	-	-
d240 (n=98)	0.52	.13	1.54	1.41	0.59	.13	1.58	1.44	0.70	.13	1.64	1.49
d410 (n=100)	-0.09	.11	1.17	1.02	-0.02	.11	1.16	1.00	0.07	.11	1.17	1.02
d415 (n=100)	-0.77	.11	0.95	0.89	-0.72	.11	0.94	0.88	-0.65	.11	0.92	0.86
d430 (n=97)	0.15	.12	1.47	1.25	0.21	.12	1.47	1.26	0.31	.12	1.48	1.26
d450 (n=100)	-0.63	.11	1.00	0.96	-0.58	.11	1.01	0.97	-0.50	.11	1.03	1.00
d455 (n=100)	-0.68	.11	0.43	0.47	-0.63	.11	0.43	0.48	-0.55	.11	0.44	0.49
d470 (n=97)	0.32	.12	0.61	0.57	0.39	.12	0.60	0.57	0.49	.13	0.62	0.59
d540 (n=98)	-	-	-	-	-	-	-	-	-	-	-	-
d850 (n=49)	-0.49	.16	1.16	1.08	-0.42	.16	1.16	1.08	-0.32	.16	1.20	1.11
d920 (n=85)	-0.70	.12	0.86	0.82	-0.65	.12	0.85	0.81	-0.57	.12	0.85	0.82
e150 (n=100)	1.46	.16	1.59	1.03	1.55	.17	1.61	1.07	1.67	.17	1.68*	1.10
Mean	0.00	.12	1.04	1.02	0.00	.12	1.04	1.01	0.00	.12	1.04	1.00
SD	0.74	.02	0.35	0.33	0.72	.02	0.36	0.32	0.65	.02	0.36	0.30

Table 1. Estimates of difficulty and adjustment to Rasch model of the comprehensive core set for knee dysfunction applied face-to-face via interview conducted by a researcher trained in the use of ICF (continued)

	**MODEL 10**				**MODEL 11**				**MODEL 12[ft]**			
Person Separation Reliability Index	2.83 (0.89)				2.85 (0.89)				2.68 (0.88)			
Item Separation Reliability Index	3.85 (0.94)				3.74 (0.93)				3.74 (0.93)			
Raw variance explained by measures	54.3%				56.2%				55.5%			
**ICF Category**	**Measure**	**SE**	**Infit**	**Outfit**	**Measure**	**SE**	**Infit**	**Outfit**	**Measure**	**SE**	**Infit**	**Outfit**
b134 (n=100)	-	-	-	-	-	-	-	-	-	-	-	-
b235 (n=100)	0.66	.12	0.80	0.94	0.75	.13	0.87	1.01	0.70	.13	0.84	0.98
b260 (n=100)	-	-	-	-	-	-	-	-	-	-	-	-
b280 (n=100)	-0.90	.11	1.33	1.40	-0.88	.11	1.37	1.43	-0.88	.11	1.33	1.39
b530 (n=100)	0.18	.12	1.15	1.39	0.24	.12	1.23	1.47	0.20	.12	1.19	1.39
b710 (n=97)	-	-	-	-	-	-	-	-	-	-	-	-
b715 (n=100)	-	-	-	-	-	-	-	-	-	-	-	-
b730 (n=99)	-0.09	.11	0.99	1.05	-0.04	.12	1.09	1.15	-0.06	.11	1.08	1.13
b760 (n=100)	-	-	-	-	-	-	-	-	-	-	-	-
b770 (n=98)	0.29	.12	0.52	0.54	0.36	.12	0.54	0.55	0.32	.12	0.51	0.51
b780 (n=97)	0.60	.13	1.30	1.17	0.68	.13	1.42	1.28	0.63	.13	1.34	1.22
s7500 (n=100)	-	-	-	-	-	-	-	-	-	-	-	-
s7501 (n=100)	-0.65	.11	0.72	0.74	-0.62	.11	0.76	0.80	-0.63	.11	0.74	0.78
s7502 (n=96)	-	-	-	-	-	-	-	-	-	-	-	-
d240 (n=98)	0.82	.13	1.61*	1.47	-	-	-	-	-	-	-	-
d410 (n=100)	0.18	.12	1.21	1.06	0.24	.12	1.19	1.04	0.20	.12	1.16	1.01
d415 (n=100)	-0.55	.11	0.92	0.86	-0.52	.11	0.91	0.85	-0.53	.11	0.88	0.81
d430 (n=97)	0.43	.12	1.50	1.29	0.50	.12	1.49	1.29	0.46	.12	1.42	1.23
d450 (n=100)	-0.40	.11	1.05	1.02	-0.36	.11	1.05	1.03	-0.38	.11	1.00	0.98
d455 (n=100)	-0.45	.11	0.45	0.47	-0.42	.11	0.46*	0.49	-	-	-	-
d470 (n=97)	0.60	.13	0.63	0.60	0.68	.13	0.63	0.59	0.63	.13	0.62	0.59
d540 (n=98)	-	-	-	-	-	-	-	-	-	-	-	-
d850 (n=49)	-0.23	.16	1.26	1.18	-0.18	.16	1.27	1.18	-0.21	.16	1.20	1.11
d920 (n=85)	-0.47	.12	0.88	0.85	-0.43	.12	0.89	0.87	-0.44	.12	0.87	0.84
e150 (n=100)	-	-	-	-	-	-	-	-	-	-	-	-
Mean	0.00	.12	1.02	1.00	0.00	.12	1.01	1.00	0.00	.12	1.01	1.00
SD	0.52	.01	0.33	0.30	0.51	.01	0.31	0.30	0.50	.01	0.27	0.26

ICF: International Classification of Functioning, Disability and Health; n: answers without the qualifiers 8 or 9; SE: standard error; SD: standard deviation; *Category excluded for not meeting the requirements of the model; -Category previously excluded; [ft]Model selected for subsequent analyses.
