# Supplementary material for: Development of a brief core set for knee dysfunction based on the International Classification of Functioning, Disability and Health: assessing construct validity and measurement potential
Source: BMC Musculoskelet Disord. 2024 Jul 3;25:512. doi: 10.1186/s12891-024-07635-3 (PMC11221104; doi:10.1186/s12891-024-07635-3)
Supplement: Supplementary file 2 — Appendix B [file 12891_2024_7635_MOESM2_ESM.pdf]

Appendix B. Estimates of difficulty and adjustment to Rasch model of the remote self-administered comprehensive core set for knee dysfunction

Table 1. Estimates of difficulty and adjustment to Rasch model of the remote self-administered comprehensive core set for knee dysfunction

	**MODEL 1**				**MODEL 2**				**MODEL 3**[π]			
Person Separation Reliability Index	2.04 (0.81)				2.10 (0.82)				2.10 (0.82)			
Item Separation Reliability Index	5.34 (0.97)				5.48 (0.97)				5.48 (0.97)			
Raw variance explained by measures	43.0%				44.6%				42.9%			
**ICF Category**	**Measure**	**SE**	**Infit**	**Outfit**	**Measure**	**SE**	**Infit**	**Outfit**	**Measure**	**SE**	**Infit**	**Outfit**
b134 (n=97)	-1.31	.12	1.04	1.06	-1.34	.13	1.06	1.09	-1.17	.13	1.05	1.09
b235 (n=99)	0.02	.15	0.85	0.82	.004	.15	0.87	0.83	0.20	.15	0.87	0.83
b260 (n=97)	1.74	.28	1.04	1.12	1.78	.28	1.04	1.11	1.95	.28	1.04	1.11
b280 (n=100)	-2.26	.12	0.99	1.00	-2.31	.12	1.07	1.08	-2.15	.12	1.07	1.08
b530 (n=99)	-0.72	.13	1.29	1.26	-.073	.13	1.33	1.28	-0.56	.13	1.32	1.28
b710 (n=97)	-0.99	.13	1.15	1.11	-1.01	.13	1.17	1.13	-0.84	.13	1.17	1.13
b715 (n=100)	-0.59	.13	0.86	0.87	-.060	.14	0.89	0.90	-0.43	.14	0.89	0.90
b730 (n=99)	-1.44	.12	0.99	1.02	-1.48	.12	1.02	1.04	-1.31	.12	1.02	1.04
b760 (n=100)	0.95	.20	0.99	1.02	.098	.20	1.02	1.04	1.15	.20	1.02	1.04
b770 (n=98)	0.40	.17	0.56	0.56	.042	.17	0.56	0.56	0.59	.17	0.56	0.56
b780 (n=100)	-0.58	.13	0.77	0.79	-0.58	.14	0.82	0.84	-0.41	.14	0.82	0.84
s7500 (n=95)	0.89	.20	1.39	0.96	.092	.20	1.43	0.98	1.09	.20	1.44	0.98
s7501 (n=96)	-0.59	.14	1.30	1.27	-0.60	.14	1.37	1.34	-0.43	.14	1.37	1.34
s7502 (n=99)	0.13	.16	1.50	1.70*	-	-	-	-	-	-	-	-
d240 (n=96)	-0.62	.14	0.96	0.94	-0.62	.14	1.02	1.00	-0.46	.14	1.02	1.00
d410 (n=100)	-0.37	.14	0.80	0.75	-0.37	.14	0.79	0.75	-0.20	.14	0.79	0.75
d415 (n=97)	-1.05	.13	1.04	1.00	-1.07	.13	1.05	1.01	-0.91	.13	1.05	1.00
d430 (n=100)	0.51	.17	1.17	1.27	0.54	.17	1.19	1.36	0.70	.17	1.19	1.36
d450 (n=100)	-0.04	.15	1.22	1.10	-0.03	.15	1.22	1.11	0.14	.15	1.22	1.11
d455 (n=100)	-0.49	.14	0.59	0.58	-0.49	.14	0.59	0.58	-0.32	.14	0.59	0.58
d470 (n=100)	1.26	.22	0.82	0.67	1.30	.22	0.82	0.66	1.46	.22	0.82	0.66
d540 (n=100)	0.99	.20	1.19	1.05	1.02	.20	1.20	1.03	1.19	.20	1.21	1.06
d850 (n=75)	0.59	.21	1.19	0.84	0.60	.21	1.21	0.85	0.77	.21	1.21	0.85
d920 (n=95)	-0.24	.15	0.97	0.93	-0.24	.15	0.99	0.95	-0.07	.15	0.98	0.95
e150 (n=100)	-1.31	.12	1.04	1.06	3.87*	.71	0.98	0.76	-	-	-	-
Mean	0.00	0.18	1.03	0.98	0.00	.18	1.03	0.97	0.00	.16	1.03	0.98
SD	1.20	0.11	0.23	0.24	1.25	.12	0.22	0.21	0.97	.04	0.22	0.21

ICF: International Classification of Functioning, Disability and Health; n: answers without the qualifiers 8 or 9; SE: standard error; SD: standard deviation; *Category excluded for not meeting the requirements of the model; -Category previously excluded; [π]Model selected for subsequent analyses.
