# Supplementary material for: Development of a brief core set for knee dysfunction based on the International Classification of Functioning, Disability and Health: assessing construct validity and measurement potential
Source: BMC Musculoskelet Disord. 2024 Jul 3;25:512. doi: 10.1186/s12891-024-07635-3 (PMC11221104; doi:10.1186/s12891-024-07635-3)
Supplement: Supplementary file 3 — Appendix C [file 12891_2024_7635_MOESM3_ESM.pdf]

Appendix C. Brief core set for knee dysfunction

BRIEF CORE SET FOR KNEE DYSFUNCTION

This core set is an instrument for assessing and measuring the functioning of people with any knee problem, based on the International Classification of Functioning, Disability and Health (ICF). This instrument' must be applied face-to-face via an interview conducted by a professional previously trained in the use of the ICF. Responses should be based on the patient's complaint at the time of evaluation. The professional must ask the patient to classify each ICF category on a scale ranging from 0 to 4, with 0 being no impairment/difficulty and 4 complete impairment/difficulty. Impairment is represented by problems in body function or structure and difficulty is represented by limitations in performing an activity or restrictions in the involvement in daily life situations[1].

**ICF CATEGORY**	No impairment 0-4%	Mild impairment 5-24%	Moderate impairment 25-49%	Severe impairment 50-95%	Complete impairment 96-100%	Not specified or not applicable
**b235 "Vestibular functions" (balance)**<br>Sensory functions of the inner ear related to position, balance and movement.<br>*Inclusions*: functions of position and positional sense; functions of balance of the body and movement.	0	1	2	3	4	X
**b280 "Sensation of pain"**<br>Sensation of unpleasant feeling indicating potential or actual damage to some body structure.<br>*Inclusions*: sensations of generalized or localized pain, in one or more body part, pain in a dermatome, stabbing pain, burning pain, dull pain, aching pain; impairments such as myalgia, analgesia and hyperalgesia.	0	1	2	3	4	X
**b530 "Weight maintenance functions"**<br>Functions of maintaining appropriate body weight, including weight gain during the developmental period.<br>*Inclusions*: functions of maintenance of acceptable Body Mass Index (BMI); and impairments such as underweight, cachexia, wasting, overweight, emaciation and such as in primary and secondary obesity.	0	1	2	3	4	X

**b730 "Muscle power functions"**<br>Functions related to the force generated by the contraction of a muscle or muscle groups.<br>*Inclusions*: functions associated with the power of specific muscles and muscle groups, muscles of one limb, one side of the body, the lower half of the body, all limbs, the trunk and the body as a whole; impairments such as weakness of small muscles in feet and hands, muscle paresis, muscle paralysis, monoplegia, hemiplegia, paraplegia, quadriplegia and akinetic mutism.	0	1	2	3	4	X
**b780 "Sensations related to muscles and movement functions"**<br>Sensations associated with the muscles or muscle groups of the body and their movement.<br>*Inclusions*: sensations of muscle stiffness and tightness of muscles, muscle spasm or constriction, and heaviness of muscles.	0	1	2	3	4	X
**s7501 "Structure of lower leg"**<br>*Inclusions*: Bones of lower leg, knee joint, muscles of lower leg, ligaments and fasciae of lower leg.	0	1	2	3	4	X
**ICF CATEGORY**	No difficulty 0-4%	Mild difficulty 5-24%	Moderate difficulty 25-49%	Severe difficulty 50-95%	Complete difficulty 96-100%	Not specified or not applicable
**d410 "Changing basic body position"**<br>Getting into and out of a body position and moving from one location to another, such as getting up out of a chair to lie down on a bed, and getting into and out of positions of kneeling or squatting.<br>*Inclusions*: changing body position from lying down, from squatting or kneeling, from sitting or standing, bending and shifting the body's centre of gravity.	0	1	2	3	4	X
**d415 "Maintaining a body position"**<br>Staying in the same body position as required, such as remaining seated or remaining standing for work or school.<br>*Inclusions*: maintaining a lying, squatting, kneeling, sitting and standing position.	0	1	2	3	4	X
**d430 "Lifting and carrying objects"**<br>Raising up an object or taking something from one place to another, such as when lifting a cup or carrying a child from one room to another.<br>*Inclusions*: lifting, carrying in the hands or arms, or on shoulders, hip, back or head; putting down.	0	1	2	3	4	X
**d470 "Using transportation"**<br>Using transportation to move around as a passenger, such as being driven in a car or on a bus, rickshaw, jitney, animal-powered vehicle, or private or public taxi, bus, train, tram, subway, boat or aircraft.<br>*Inclusions*: using human-powered transportation; using private motorized or public transportation.	0	1	2	3	4	X
**d850 "Remunerative employment"**<br>Engaging in all aspects of work, as an occupation, trade, profession or other form of employment, for payment, as an employee, full or part time, or self-employed, such as seeking employment and getting a job, doing the required tasks of the job, attending work on time as required, supervising other workers or being supervised, and performing required tasks alone or in groups.<br>*Inclusions*: self-employment, part-time and full-time employment,	0	1	2	3	4	X

**d920 "Recreation and leisure"**<br>Engaging in any form of play, recreational or leisure activity, such as informal or organized play and sports, programmes of physical fitness, relaxation, amusement or diversion, going to art galleries, museums, cinemas or theatres; engaging in crafts or hobbies, reading for enjoyment, playing musical instruments; sightseeing, tourism and travelling for pleasure.<br>*Inclusions*: play, sports, arts and culture, crafts, hobbies and socializing.	0	1	2	3	4	X

$$\text{Score} = \frac{\text{(sum of the qualifiers)}}{\text{(number of categories answered)}} \times 25$$

Scoring:

0-4: no functioning compromise

5-24: mild functioning compromise

25-49: moderate functioning compromise

50-95: severe functioning compromise

96-100: total functioning compromise

Reference

1. World Health Organization. *International Classification of Functioning, Disability and Health: ICF*. World Health Organization; 2001.
